# Effects of affiliation-, achievement-, and power-related topics in mathematical word problems on students’ performance, task-related values, and expectancies

**DOI:** 10.1371/journal.pone.0270116

**Published:** 2022-06-30

**Authors:** Bettina Scheidemann, Hedwig Gasteiger, Rosa M. Puca

**Affiliations:** 1 Department of Educational Psychology, Institute of Psychology, Osnabrück University, Osnabrück, Germany; 2 Department of Mathematics Education, Institute of Mathematics, Osnabrück University, Osnabrück, Germany; East China Normal University, CHINA

## Abstract

A motivational downturn in mathematics during secondary school has been well documented for many students. As a way to address this, creating personally relevant tasks is supposed to increase students’ motivation for mathematical tasks. According to recent research, topics relating to affiliation, achievement, and power are personally relevant for young people. Prior research showed that motive imagery in school tasks increases students’ task-related intrinsic value and success expectancies. The present study examined the effect of motive topics in word problems on students’ task performance. We contextualized mathematical tasks either with affiliation, achievement, and power topics or with neutral topics not related to motive topics. The tasks were comparable regarding their mean word count, text, and mathematical complexity. In three experimental studies (*N*_1_ = 56, *N*_2_ = 63, *N*_3_ = 62), the students solved four tasks for each motive topic and neutral tasks, respectively. The dependent variables were task performance, intrinsic values, and expectancies of success. Repeated measures analyses of variance with the four-level, within-subjects factor motive imagery revealed positive effects of motive imagery in word problems on students’ task performance. This was particularly true for achievement-related tasks compared with neutral tasks. The results also indicated slightly positive effects for affiliation-related word problems on students’ performance. In addition, the intrinsic value for affiliation-related (Experiment 1) and achievement-related tasks (Experiment 3) was higher than for neutral tasks. Power imagery did not affect students’ task performance; it negatively affected students’ intrinsic value compared with neutral tasks. Task-related success expectancies were not influenced by motive imagery. The present study replicates and extends previous findings that indicate that tasks referring to affiliation and achievement imagery are more appealing to students and can benefit their performance.

## Introduction

It is a frequently observed phenomenon that students’ learning motivation and interest declines over their academic trajectory (e.g., [1–3]). This decrease in interest and motivation is most pronounced in secondary school (e.g., [1, 2, 4–6]) and is often accompanied by poorer school performance [1, 7]. Mathematics is a subject that seems to be particularly affected by this negative development. Several studies have shown a decline in mathematical interest, learning motivation, and enjoyment during the course of school [4–6, 8–12]. This downward trend is worrying because motivation is a very important predictor for mathematical achievement [13]. For the motivational construct of interest, research has shown direct and indirect (via academic engagement, persistence, or course selection) effects on mathematics achievement [14–17]; here, interest in mathematics was found to be an important predictor for mathematics achievement in the long term [18]. Given these findings, it seems necessary to closely monitor students’ interest so that a response can be issued when their interest is low. However, the question remains regarding how to increase students’ motivation for mathematics if they are not interested in the subject matter itself.

### How to increase students’ motivation?

According to the expectancy-value model of achievement motivation, people’s motivation arises from their expectation of success for a task or activity and the subjective value of this task or activity [19–21]. Expectations of success are defined as people’s beliefs about how well they will accomplish an upcoming task. Subjective task values are further distinguished into three components that are important for learning motivation: attainment value (importance to do well), utility value (usefulness of the task, (e.g., for attaining future goals), and intrinsic value. Intrinsic value is described as “the subjective interest the individual has in the subject” [20, p. 120] or “the enjoyment one gains from doing the task” [21, p. 72].

Besides performance, one focus of our research is the intrinsic value of tasks. Closely related to intrinsic value is the motivational construct of interest (e.g., [22]). Although these two constructs have distinct theoretical roots, they overlap to some extent (cf. [20, 21]), providing a theoretical basis for our research approach. Researchers have distinguished between individual interest and situational interest [22–24]. *Individual interest* is defined as a persons’ relatively stable orientation toward a certain topic, object, material, or activity [22, 24, 25]. As outlined before, individual interest (e.g., in mathematics) positively affects students’ engagement, persistence, and performance [15–17, 24, 26]. In contrast, *situational interest* is conceptualized as a temporary emotional state [27, 28] and is closely related to the construct of intrinsic value [21, 29]. It involves “focused attention, increased cognitive functioning, persistence, and affective involvement” [28, p. 32] and can also positively influence learning [25]. According to interest research, situational interest is context specific. Engaging the features of a lecture, text, or task can arouse situational interest [20, 22, 23, 27, 30, 31], even if students’ individual interest is actually low.

#### Features of engaging, interest-evoking tasks

Engaging features relate to formal structural task characteristics such as novelty, complexity, or ambiguity [32], as well as to personally relevant or meaningful contents with which the learner can identify. Regardless of the school subject, student’s interest can be increased if the materials or lessons are relevant for students’ needs and goals [33] or related to their life themes, experiences, or specific interests [26, 27, 34–39].

According to these suggestions and referred research findings, situational interest and intrinsic value for mathematical tasks could increase if the tasks refer to students’ personal experiences, life themes, needs, or goals with which students can identify.

In sum, personally relevant and meaningful contextual information can increase situational interest and promote intrinsic task values, which is an important aspect of the expectancy-value model of achievement motivation. Within the expectancy-value model of achievement motivation, task-related expectancies and values are theoretically distinct. Nevertheless, they are positively related to each other [20]. Moreover, expectancies and values are assumed to affect achievement-related choices, effort, persistence, and performance [19–21]. Thus, with reference to the expectancy-value model of achievement motivation, personally relevant task contexts could enhance not only students’ subjective task values, but also students’ task-related success expectancies, and they can also positively affect students’ performance. Some findings on contextualized math tasks suggest that students can benefit from personally relevant task contexts [e.g., 40]. In the following section, we present research on task contextualization.

#### Research on task contextualization

Mathematical tasks can be more or less connected to real-life issues [41–43]. In the research on task contextualization in mathematics, two different task types can be distinguished: intramathematical problems (“pure” number problems) and contextualized problems with reference to reality (e.g., word problems), or even real problems. There are several reasons for embedding mathematical problems in reality-related contexts, such as enhancing students’ transfer between mathematical practice in school and the real world, increasing students’ accessibility because of familiar, concrete, and imaginable situations, and to arouse interest and motivate students [43–46].

In addition to the different degrees of realism, mathematical problem types also differ regarding the mental activities required to solve them (e.g., [42]). Working on contextualized problems requires situational understanding, a demanding transformation process, and more reading comprehension than working on intramathematical tasks [47]. Nevertheless, there is empirical evidence that students perform better in contextualized problems compared with intramathematical problems with corresponding mathematical procedures [40, 45, 47, 48]. The verbal context is assumed to help students recognize and imagine the mathematical operations, thus improving the accessibility of the problem [45, 47]; this can evoke the use of different strategies more than working with pure number problems would [45].

However, research has produced mixed results regarding the effects of reality-related task contexts on students’ motivation and performance. Although Pekrun et al. [11] found that students reported more enjoyment for tasks with a reference to reality than for bare number problems without, other studies found no differences in students’ enjoyment, interest and task value between realistic tasks and those without relation to reality [41, 42]. Regarding students’ task performance, there are studies that have compared different task contexts with each other. In these studies, only contextualized tasks (e.g., dressed-up word problems) were used, and the contexts differed in terms of their personal relevance. It was found that task contexts that were individualized or personalized, either by students’ personal background information (e.g., friends’ name) or by their out-of-school interests, which positively affected students’ motivation and learning [40, 49–51]. For instance, within the experiment by Cordova and Lepper [40], students worked with a computer-based learning game in which the material was presented abstractly (control condition), embedded in fantasy contexts (contextualized condition), or embedded in fantasy contexts that were embellished with personal background information (personalized condition). Those students who worked in personalized learning contexts showed more enjoyment, task involvement, and greater learning compared with students in the contextualized but not personalized condition or in the control condition [40]. In contrast to these positive effects of reality-related task context on performance, there are also research findings pointing in a different direction, indicating that a lifelike, familiar task context is not supportive per se. It has been shown that a familiar context can hinder finding a successful solution because the context was lifelike but negatively connoted for the student who solved the task [45, p. 8, 52].

Even though the research in this area is complex and not always clear, there is some evidence indicating that mathematical tasks within those contexts relating to students’ personal experiences, interests, or needs can increase their motivation and learning. This should be particularly true if the task contexts are positively connoted for students. However, students’ specific interests and personal information are very heterogeneous, and selecting or developing different tasks according to individual interests may be difficult to put into practice. Instead of individualizing the task context, we see the possibility of creating tasks by referring to only a few personally relevant topics that can be derived from developmental psychology, motivational psychology, and related research findings.

#### Personally relevant topics for students

In their (early) adolescence, students experience many developmental changes and must cope with new developmental tasks [2, 7]. The psychological “needs to achieve competence, autonomy, and relatedness” [7, p. 31] play an important role during this time. A central task for (early) adolescents is to develop their own identities. Young people seek independence from their parents, while other social issues such as peer acceptance become increasingly important [1, 2, 7, 53]. Since 1953, the Shell-Youth Survey has assessed life values among German youth aged between 12 and 25 years. The results of the latest Shell-Youth Survey confirmed the importance of affiliation-related issues (e.g., friendships, relationships, and family) for adolescents and young adults [54]. In addition, values relating to achievement (e.g., being ambitious and hardworking) or power and prestige (e.g., having power and influence, a high standard of living, asserting one’s own needs against others) were also rated as important [54]. A similar pattern appears for German students who are in their twenties. Most important for students were social issues (family, friends, social environment), followed by career opportunities and material well-being [55]. These findings imply that affiliation-, achievement-, and power-related topics are personally relevant and potentially motivating for adolescents and young adults. It could be assumed that students feel personally connected to tasks relating to affiliation-, achievement-, and power-related topics and are motivated to deal with them. These relevant issues correspond to the motive-specific classes of incentives described by McClelland [56]. In motivational psychology, the most frequently examined incentive classes are friendly, social relationships, establishing, and maintaining social contacts (affiliation); mastering challenges, achieving, or exceeding quality standards (achievement); and influencing or impressing other people, status, and prestige (power). According to McClelland [56], we refer to affiliation-, achievement-, and power-related topics as “motive imagery.” So far, only a few studies have dealt with motive imagery in school tasks or schoolbooks. In the following section, we provide an overview of the research in this area.

### Motive imagery in textbooks and school tasks

Engeser, Rheinberg, and Möller [57] analyzed German textbooks regarding their motive-related content. By coding second-and ninth-grade textbooks from two German federal states, the authors tested McClelland’s [58] hypothesis that achievement imagery in texts (e.g., textbooks, speeches) is an indicator of the motivational climate in a society. Affiliation, achievement, and power imagery were coded with Winter’s [59] “Manual for Scoring Motive Imagery in Running Text.” The results confirmed the assumption that textbooks from the state with better economic and educational conditions contained more achievement imagery. Additionally—and most importantly for our research—it has been shown that second- and ninth-grade textbooks differed from each other regarding their motive-related content. Although affiliation-related topics dominated in second-grade textbooks, ninth-grade textbooks barely contained these contents. In these textbooks, achievement- and power-motive imagery dominated. Given the fact that all motive-related topics are potentially relevant for students, the question arises if and to what extent the topics in textbooks and tasks meet students’ interests or needs.

A recent study with fifth-grade students took up these questions by investigating to which kind of motive-related topics secondary school students are actually most attracted [60]. For this purpose, mathematical word problems and essay tasks of representative German textbooks were enriched with affiliation, achievement, and power imagery or to none of them (neutral tasks). The authors conducted four classroom experiments with fifth-grade students. Within two experiments, the students rated intrinsic task value of the tasks (how much they like to work on it), and in two experiments, the students evaluated their expectation of success in the tasks (how confident they were in solving it). The tasks were parallelized regarding their mean word account, emotional connotations, and suspected difficulty. It was shown that fifth graders were more attracted to motive-related tasks than to neutral tasks (tasks not referring to motive imagery). Moreover, they were more confident in solving motive-related tasks than neutral ones. These positive effects were particularly found for tasks relating to affiliation-motive imagery, followed by achievement-related tasks and power-related tasks.

Hence, motive imagery in school tasks can increase students’ tasks related self-efficacy expectations and intrinsic task values, thus promoting students’ motivation. This particularly applies for tasks enriched with affiliation-motive imagery because these tasks were superior to the neutral tasks in all of the presented experiments. Nevertheless, some of the experiments also revealed positive effects for achievement and power imagery on students’ task values and expectancy beliefs so that these topics have the potential to be motivationally relevant, too. To date, it is still an open question whether motive imagery in tasks not only foster students’ expectancies and task values, but also increase students’ performance. The present research addresses this issue.

## The present research

In our research, we investigate whether affiliation-, achievement-, and power-related tasks can enhance students’ motivation and performance. As an indicator of motivation, we focus on intrinsic task values and task-related success expectancies, here following the expectancy-value model of achievement motivation [20, 21].

From a developmental psychological perspective and according to recent research findings [7, 54, 55], motive imagery is assumed to be personally relevant to students; thus, students should feel personally connected to tasks relating to these topics. Consequently, we propose that the tasks with these topics are more attractive for young people than neutral tasks. In line with this assumption, previous findings have shown that motive imagery in tasks increased students’ task-related expectancies and intrinsic task values [60].

The main goal of the present study is to investigate the influence of motive imagery in mathematical tasks on students’ task performance. According to the expectancy-value model of achievement motivation [20, 21], enhancing task-related values and expectancies could also increase students’ performance in the corresponding tasks. The findings on task individualization (e.g., [47, 51]) support this assumption.

Accordingly, we expect students to show greater performance in motive-related math tasks than in math tasks without these topics (neutral tasks). Besides the influence of motive imagery in tasks on students’ performance, we have also aimed to replicate the previous findings that have shown positive effects of motive imagery in school tasks on students’ task-related values and success expectancies when compared with neutral tasks [60]. By additionally assessing students’ task-related values and expectancies, we were also interested in exploring the possible relations between students’ task evaluation and performance in the corresponding tasks. According to the expectancy-value model and related findings, increased intrinsic task values and success expectancies should lead to increased motivation to engage in the corresponding tasks, thus potentially influencing performance [19–21]. Thus, we theoretically expect that high task values and expectancies are positively related to better task performance in corresponding tasks.

The present research addresses important issues regarding the motivational function of motive imagery in school tasks and its influence on students’ task performance. Given the findings of declining learning motivation and (mathematical) interest over the course of school [e.g., 1, 2, 4, 6, 8, 11], the motivational function of instructional materials, such as school tasks, is particularly relevant.

Affiliation, achievement, and power imagery do appear in textbooks and tasks [57]; however, their effects on students’ motivation and performance have rarely been studied. By examining this, the present research findings provide important theoretical and practical implications for making school tasks more appealing and motivating for students, which may increase their learning and performance. In particular, the use of only a few personally relevant topics—referring to students’ motivational needs for affiliation, achievement, and power—would provide a practical alternative to individually personalize tasks and materials in (mathematics) instruction.

### Materials and design

We took mathematical tasks from a representative study on civic competence in numeracy [61] and contextualized them with motive imagery. The study on civic competence in numeracy was commissioned by “Stiftung Rechnen,” in cooperation with the weekly newspaper *DIE ZEIT*, Martin Luther University of Halle-Wittenberg, and Saarland University; the study was conducted by forsa [61, 62]. Within the study, 1,027 adults in Germany aged between 18 and 65 years were tested regarding their mathematical skills in everyday contexts. The test consisted of lifelike tasks covering the basic mathematical fields from elementary to middle school (e.g., simple arithmetic, geometry, fractions; see [62] for the original test). For the present study, we initially chose four types of tasks—one task from each of the following mathematical topics: “simple arithmetic,” “direct and inverse proportionality,” “geometry,” and “fractional arithmetic.” The criteria for task selection were task difficulty and that the tasks were suitable for reformulation. We chose those tasks that had been answered correctly by 52–81% of the participants in the original sample (cf. [62]). Thus, the task difficulty was medium to rather easy.

In the present experiments, the tasks were contextualized with motive topics; motive imagery was systematically varied in within-subject designs. We implemented a within-subject design to minimize the influence of person-related confounding variables, as well as for economic reasons. To contextualize the tasks, we reformulated each of the four initially chosen tasks so that they were related either to achievement, affiliation, or power topics or to none (neutral tasks). That is, each of the four tasks was quadrupled.

Thus, there were 16 mathematical word problems in total, with four tasks for each type of motive imagery and neutral category. Within all word problems, the motive-related and neutral context information was irrelevant for solving the task but was assumed to be motivating due to its personal relevance or served as a control condition in neutral tasks, respectively.

We used Winter’s [59] *Manual for Scoring Motive Imagery in Running Text* as a guideline for the formulation of the motive-related tasks to avoid instinctive task formulation. The manual has often been used to assess affiliation, achievement, and power imagery in running texts like schoolbook excerpts, children’s books, and tasks or political speeches and has been validated for different applications (e.g., [63–66]). According to Winter [59], affiliation imagery is scored when a text contains those issues regarding the establishment, maintenance, or restoration of friendly relations among people, such as the expression of positive, friendly feelings toward persons and nations, negative feelings about separation, friendly and companionable activities, and supportive and friendly acts [59, pp. 12–14]. The achievement imagery in a text is expressed by issues regarding a standard of excellence, such as adjectives that positively evaluate performances, a positive evaluation of goals or performance, winning, or competing with others, negative feelings about failure or the lack of excellence, and unique accomplishments [59, pp. 8–11]. Power imagery is scored when a text contains issues regarding the impact, control, or influence on another person, group, institution, or country, such as strong and forceful actions, control, or regulation, attempts to influence, persuade, and convince, giving unsolicited advice or help, impressing others and mentions of prestige and fame, and triggering strong emotional reactions in a person, group, or nation [59, pp. 15–18]. The emotional reactions triggered must be intended and can be either positive (e.g., joy, enthusiasm) or negative (e.g., sadness, fear).

Two independent persons who were familiar with Winter’s manual but not involved in task creation coded the tasks with respect to the four categories (affiliation, achievement, power, and neutral). For the experiments, we used only tasks that had been rated unambiguously as achievement, affiliation, or power related or neutral. Within each mathematical topic (e.g., simple arithmetic), the mathematical tasks differed regarding their motive imagery but were comparable regarding relevant features, such as their mean word count (neutral tasks *M* = 66.25, *SD* = 11.90; affiliation imagery *M* = 67.25, *SD* = 11.44; achievement imagery *M* = 66.50, *SD* = 11.62; power imagery *M* = 67.75, *SD* = 10.34), text complexity, suspected difficulty, and emotional connotation, as well as the protagonists’ name and gender. In total, there were two female and two male protagonists. The sample tasks are shown in Table 1. We used a text analysis online tool [67] to determine the text complexity of the 16-word problems. The text analysis tool generates a score (readability index) to determine the comprehensibility of a text, ranging from “very demanding” (0–20) to “very easy” (81–100). The average index for all motive-related and neutral word problems used within the present experiments was “easy to understand” (61–80). Within each mathematical topic, the mathematical operations (e.g., multiplication, addition), numerical range, and number of calculation steps needed to solve the tasks were held constant to ensure comparable task difficulty.

**Table 1:** Sample tasks for the mathematical topic “direct and inverse proportionality”.

Motive imagery	Sample task
Affiliation	Sabine and her two flatmates would like to renovate the basement room of their shared flat to use it as a living and party room in the future. Because they get on well with each other and it is more fun, they also go shopping together. They decide on yellow wall paint. The basement room measures **5.50 m x 4 m,** and the walls are **2 m** high. A **4-liter** bucket of the wall paint is enough for **20 m^2^**. How many paint buckets should you buy if you want to paint all the walls and ceiling?





Achievement	Sabine is currently training as a painter. She is very hardworking, and in her vocational school class, she is top of her class. For this reason, at an early stage, her boss assigned her the responsible task of painting a customer’s room white all by herself. The customer’s windowless room measures **6 m x 3 m,** and the walls are **2.50 m** high. A **10**-**liter** bucket of the white paint is enough for **40 m**^2^. How many paint buckets does Sabine need if she has to paint all the walls and ceiling of the room?





Power	According to the rental contract, Sabine has to hand over her apartment completely renovated to the next tenant after a move that she is currently planning. When the owner comes to view the apartment, Sabine defends herself against the claim. She can convince him that only one of the rooms is in need of renovation. The windowless room measures **4 m x 3 m** and is **3.50 m** high. A **2-liter** bucket of the cheapest paint is enough for **10 m^2^.** How many buckets of paint does Sabine have to buy if she needs to paint all the walls and ceiling of the room?






Neutral	Shortly after her moving, Sabine decides to paint the windowless basement room in her new apartment. In the construction market, she stands in front of the shelf with the different wall colors. She thinks about it briefly and finally decides on a simple white color. The basement room she wants to paint measures **6 m x 4 m,** and the walls are **2.50 m** high. A **5-liter** bucket of the white wall paint is enough for **30 m^2^.** How many paint buckets should Sabine buy if she wants to paint all the walls and ceiling?






Note. The tasks were originally presented in German and parallelized with respect to their mean word count. In the English translation of the tasks presented here, there are some deviations in task length (number of words and characters) that were not present in the German version. Bold: In the third experiment, the bold printed values were randomly permuted between the motive-related and neutral tasks belonging to the same mathematical topic (e.g., direct and inverse proportionality). The tasks were presented to participants without bold printed values.

### Overview of present experiments

Three experiments in which students worked on motive-related and neutral word problems were conducted to test the effect of motive imagery in mathematical word problems on students’ task performance. Following the expectancy-value model of achievement motivation [20, 21], we additionally assessed students’ task-related success expectancies and values in the first and third experiments. This gave us the opportunity to replicate previous findings [60] and examine the possible relations between students’ task evaluation and performance in the corresponding tasks. In the third experiment, we additionally assessed students’ math-related self-concept and math anxiety to investigate the relations of those constructs with students’ task evaluation and performance. For all of the present experiments, we used the same 16 motive-related and neutral word problems. However, in the third experiment, we interchanged the numerical data (cf. Table 1, bold printed values) in the tasks on the same mathematical topic to ensure that the differences between motive-related and neutral tasks cannot be explained simply by different numerical data in the tasks.

The present research complies with the ethical standards of the German Research Foundation in the treatment of human samples and adhered to the principles of the Ethics Committee of the University of Osnabrück. Consent information was included online prior to the studies. In Experiment 1, informed consent was obtained before the participants chose a suitable date online to participate in the laboratory experiment. In Experiments 2 and 3, informed consent was obtained at the beginning of the online surveys. We informed the participants that the study would be conducted completely anonymously. Furthermore, the participants were informed that the anonymized data would be used for research purposes only. It was emphasized that participants could withdraw at any time without personal consequences. The participants were informed in advance about the scope and duration of the study and tasks involved (e.g., rating and completing math tasks). The participants provided their electronic informed consent by clicking “continue” after reading respective information if they agreed and wished to participate.

The participants were not misled or misinformed about the purpose of the study. For the present study, we used material commonly used in the classroom and found in textbooks. The experimental manipulation consisted only of different task formulations, as can be found in common textbooks and that students also encounter in daily life. Thus, there was no reason to expect that the participants would be exposed to risk or harm as a result of their participation. Based on these aspects, we did not seek formal ethical approval from an ethics committee. After participation, the students were informed of the purpose of the various task formulations.

## Experiment 1

### Methods

#### Participants

A total of 58 participants (41 female) participated. They were recruited via e-mails, flyers, and notice boards at the university and other public buildings. The participants’ average age was 25.82 (*SD* = 8.43), ranging from 18 to 60 years. The majority of the sample (85%) were students at a German university. They studied different subjects. The average last math grade in high school was 2.47 (*SD* = 1.04). German grades range from 1 (very good) to 6 (insufficient). The participants received 10 euros for their participation.

#### Measures and procedure

The participants received further information about the study via the internet and selected suitable appointments online to participate in the laboratory experiment. The laboratory experiment consisted of two parts and was conducted in a small and separate laboratory cabin with a time interval of one week. The participants were not allowed to take any belongings (e.g., calculator, smartphone) with them into the laboratory cabin.

During the *first part*, the participants completed a short questionnaire with demographics. The next part was programmed using the software package E-Prime [68], and the participants received further instructions via a computer screen. They were instructed to carefully read and evaluate the subsequent mathematical tasks but not to solve them. Next, the 16 motive-related and neutral word problems were consecutively presented to the participants on the screen. Examples of motive-related and neutral word problems are presented in Table 1. Each task was separately displayed for 30 seconds, and the participants evaluated the respective task on a subsequent screen page. The participants had to evaluate the tasks in two blocks, measuring intrinsic task value in the first block and self-efficacy expectations in the second block. The two blocks were balanced: half of the participants started with block A, which measured intrinsic task value for all 16 motive-related and neutral tasks, and half of the participants started with block B, which measured task-related self-efficacy expectations for all 16 motive-related and neutral tasks. The presentation of all motive-related and neutral math tasks within blocks A and B was randomized (individually for each participant). The intrinsic task value for each mathematical task was assessed by the following question: “How much do you like the task?” The participants evaluated each task (four tasks for each type of motive imagery or neutral, respectively) on a 6-point rating scale ranging from 1 = *very much* to 6 = *not at all* by entering the respective numbers on the keyboard. The participants’ self-efficacy expectations for each mathematical task were assessed by the following question: “How much time do you probably need to complete the task?” The participants were asked to enter the estimated time to complete the task in minutes and seconds. We considered this to be an indirect measurement of self-efficacy expectations, with shorter estimated processing times indicating higher self-efficacy expectations.

In the *second part* of the experiment, we assessed students’ performance in motive-related and neutral word problems. The tasks were the same as those in the first part. All tasks required basic numeracy skills that the participants should have acquired during their school years. However, some of the participants might not have been used to these kinds of tasks at this point in their lives. By implementing a training session prior to the start of the experiment, we wanted to reduce the practice effects during the actual experiment. The practice block consisted of the four original tasks from the study on civic competence in numeracy [61, 62], which served as the basis for the formulation of the 16 motive-related and neutral tasks, respectively (cf. section “Materials and design”). That is, the four training tasks each dealt with one of the mathematical topics: “simple arithmetic,” “direct and inverse proportionality,” “geometry,” and “fractional arithmetic” but were not enriched with motive imagery and contained different numbers than the 16 motive-related and neutral tasks. In doing so, the participants were familiarized with the different task types and experimental procedure on the computer.

After a short introduction via a computer screen, the participants completed the four training tasks. Next, the 16 motive-related and neutral word problems were randomly presented on the computer. For each task, the participants were asked to solve the task. They were allowed to use paper and pencil, which was provided in the laboratory cabin. The participants entered their solution into a text box on the computer screen and then continued with the next task. There was no time limit for completing the tasks. Task performance was measured by the mean number of correctly solved tasks in the four tasks on the same motive imagery (affiliation, achievement, and power) and neutral, respectively.

After task completion, a questionnaire was administered. The participants indicated their average math grade at school and whether they were familiar with the tasks or the study on civic competence in numeracy [62], respectively. Finally, the participants were asked about their presumption of the aim of the present study to check their awareness of the experimental manipulation.

### Results and discussion

We conducted repeated measures analyses of variance with the four-level within-subjects factor motive imagery to analyze the data. The different task-topics affiliation, achievement, power, and neutral were the four levels of the motive imagery factor. The dependent variables were the mean liking ratings (intrinsic task value), the mean estimated time to perform the tasks (self-efficacy expectations), and the mean number of correctly solved tasks (performance) in the four tasks on the same motive imagery (affiliation, achievement, power, and neutral), respectively. According to our theoretical assumptions, we used planned simple contrast to test the mean differences between neutral tasks and motive-related tasks. The Greenhouse–Geisser adjustment was used to correct for violations of sphericity and will be indicated in the corresponding sections, if needed. Descriptive statistics are presented in Table 2.

**Table 2:** Means and standard deviations for intrinsic task values and expectancies of success and performance in neutral tasks, and tasks related to affiliation, achievement, and power in Experiment 1.

	Intrinsic task values^1^		Expectancies of success^2^		Task performance^3^	
Motive imagery	*M*	*SD*	*M*	*SD*	*M*	*SD*
Affiliation	4.55	0.69	110.05	65.17	2.91	0.98
Achievement	4.48	0.68	106.83	69.72	3.03	0.88
Power	4.13	0.79	119.28	71.55	2.57	0.99
Neutral	4.38	0.76	111.03	66.42	2.53	1.06

Note

^1^Items were recoded so that high numbers indicate high intrinsic task values (1–6)

^2^Number of correctly solved tasks (0–4). ^2^Expected time needed to successfully perform the task (seconds); ^3^Number of correctly solved tasks (0–4).

We additionally tested for possible gender effects, finding no gender main effect or gender x motive imagery interaction on task evaluation (intrinsic task value, self-efficacy expectation) or performance (*p*s >.10). Correlations between the variables intrinsic task value, self-efficacy expectations, and performance are presented in Table 3. The correlations were in the expected directions, albeit mostly insignificant. That is, high intrinsic task values and task-related self-efficacy expectations are related to better task performance.

**Table 3:** Intercorrelations for students’ math grades in high school, intrinsic task values, and expectancies of success and performance in neutral tasks and tasks related to affiliation, achievement, and power in Experiment 1.

		Intrinsic task values^2^				Expectancies of success^3^				Task performance^4^			
		neutr.	aff	ach	pow	neutr.	aff	ach	pow	neutr.	aff	ach	pow
**math grade** ^**1**^		-.13	-.14	-.06	-.08	.22	.18	.16	.15	-.22	-.04	-.29*	-.17
**intrinsic task values**													
	neutral (neutr.)	−	.74**	.75**	.61**	-.11	-.18	-.11	-.05	.19	.08	.04	.15
	aff		−	.77**	.61**	-.14	-.19	-.17	-.13	.16	.15	.17	.10
	ach			−	.58**	-.18	-.24	-.20	-.23	.18	.17	.14	.12
	pow				−	-.30*	-.35**	-.30*	-.22	.31*	.27*	.20	.16
**expectancies of success**													
	neutr.					−	.94**	.94**	.90**	-.20	-.20	-.37**	-.23
	aff						−	.93**	.89**	-.21	-.21	-.27*	-.17
	ach							−	.91**	-.20	-.24	-.33*	-.19
	pow								−	-.19	-.21	-.29*	-.15
**performance**													
	neutr.									−	.61**	.56**	.62**
	aff										−	.57**	.52**
	ach											−	.57**
	pow												−

Note

* p < .05

** *p* < .01

*N* = 58. ^1^Gernan grading system: low numbers indicate high grades (e.g., 1 = very good, 6 = very bad).

^2^Items were recoded so that high numbers indicate high intrinsic task values and high expectancies of success, respectively.

^3^Estimated time to successfully complete the task (seconds).

^4^Number of correctly solved tasks.

#### Intrinsic task values

Mauchly’s test revealed that the assumption of sphericity had been violated for the factor motive imagery (χ2(2) = 13.74, *p* < .05); therefore, degrees of freedom were corrected using Greenhouse–Geisser estimates of sphericity (ε = 0.853). In line with our expectations, the repeated measures analysis of variance revealed a statistically significant main effect for motive imagery *F*(2.559, 145.887) = 10.78, *p* < .001, η_p_^2^ = .16. Planned simple contrasts showed that the participants liked affiliation-related tasks more than neutral tasks *F*(1, 57) = 5.87, *p* = .019, η_p_^2^ = .09. In contrast to our expectations, the participants liked power-related tasks less than neutral ones, *F*(1, 57) = 7.48, *p* = .008, η_p_^2^ = .12. There was no statistically significant difference between achievement-related and neutral tasks, *F*(1, 57) = 2.17, *p* = .146, η_p_^2^ = .04.

#### Self-efficacy expectations

Mauchly’s test revealed that the assumption of sphericity had been violated for the factor motive imagery (χ2(2) = 12.23, *p* < .05); therefore, degrees of freedom were corrected using Greenhouse–Geisser estimates of sphericity (ε = 0.862). Repeated measures analysis of variance revealed a statistically significant main effect for motive imagery, *F*(2.585, 147.351) = 4.20, *p* = .010, η_p_^2^ = .07. Planned simple contrasts showed that the participants expected to need more time to successfully complete power-related tasks than neutral tasks *F*(1, 57) = 4.11, *p* = .047, η_p_^2^ = .07. There were no statistically significant differences between affiliation-related and neutral tasks *F*(1, 57) = 0.10, *p* = .750, η_p_^2^ = .002 or between achievement-related and neutral tasks *F*(1, 57) = 1.88, *p* = .175, η_p_^2^ = .03.

#### Performance

Regarding the mean number of correctly solved tasks, repeated measures analysis of variance revealed a statistically significant main effect for motive imagery *F*(3, 171) = 9.56, *p* < .001, η_p_^2^ = .14. Planned simple contrasts showed that the participants solved more affiliation-related tasks *F*(1, 57) = 11.42, *p* = .001, η_p_^2^ = .17, as well as more achievement-related tasks *F*(1, 57) = 18.57, *p* < .001, η_p_^2^ = .25 correctly than neutral tasks. There was no statistically significant difference between power-related and neutral tasks, *F*(1, 57) = 0.9, *p* = .771, η_p_^2^ = .001.

To summarize, there were statistically significant main effects for motive imagery on intrinsic task value, self-efficacy expectation, and performance. The effects were mainly in the expected directions. However, this did not apply to all motive-related tasks. In the present experiment, the students liked *affiliation-related tasks* the most. This is in line with previous research, which has always revealed the highest intrinsic task values for affiliation-related tasks [60], thus further supporting the high relevance of social issues for adolescents and young adults [1, 2, 7, 54, 55].

Furthermore, the present experiment extends previous research findings. It was found that affiliation-motive imagery positively affected students’ performance. The same holds for *achievement-related tasks* because students’ performance in these tasks was also better than in the neutral task. In contrast to our expectations, achievement-motive imagery did not affect students’ intrinsic task value or self-efficacy expectation compared with students’ intrinsic value or self-efficacy expectations for neutral tasks. On a descriptive level, students were expected to need the shortest time to successfully complete achievement-related tasks. For *power-related tasks*, no effect was found regarding students’ performance compared with neutral tasks. Thus, power-motive imagery neither positively nor negatively affected students’ performance. Regarding task-related expectancies and values, *power-related tasks* were even inferior to neutral tasks. That is, the students liked power-related tasks less than neutral tasks and were expected to need more time for task completion compared with neutral tasks. This negative effect on task evaluation is not in line with previous research. This could be because of the decreasing importance of power-related topics for young adults compared with students in fifth grade in previous experiments [60]. However, at least for task-related self-efficacy expectations, the present findings are not fully comparable with earlier study results. In the present experiment, we did not ask the participants directly about their expectancies of success but rather about the estimated time needed for processing the tasks. Moreover, because of the free response format, there were strong variations in the data on self-efficacy expectations. This could be prevented by using a rating scale with a predefined range.

Although not all hypotheses could be confirmed, the present experiment extends earlier research results by showing that affiliation and achievement-motive imagery increased students’ performance compared with neutral tasks. The effect sizes reveal that the comparison with neutral tasks was the most pronounced for achievement-related tasks, followed by affiliation-related tasks. Because we found considerably strong effects of affiliation- and achievement-motive imagery on performance, we first wanted to replicate these findings with a new but comparable sample of students. A more detailed discussion will be given in the last section of the paper (general discussion).

## Experiment 2

### Methods

#### Participants

As in Experiment 1, the participants were recruited via e-mails, flyers, and notice boards at the university and other public buildings. A total of 70 participants participated. We excluded seven participants from analyses because they had already taken part in the first experiment. Thus, there were 63 remaining participants (49 female). Their average age was 23.30 years (*SD* = 3.51), ranging from 19 to 39 years. The average last math grade in high school was 2.48 (*SD* = 1.11). The majority (95%) were students at a German university, and they studied different subjects. They received 10 euros for participation.

#### Measures and procedure

First, the participants completed a short questionnaire with demographics via the internet and then chose a suitable date for participating in the experiment. The experiment was conducted in a small and separate laboratory cabin. This time, we were primarily interested in replicating the previous findings on the influence of motive imagery in math problems on task performance. Therefore, we only assessed students’ performance in the motive-related and neutral tasks, without measuring task-related expectancies and values. Accordingly, Experiment 2 consisted of only one part. The procedure and materials used to measure task performance were the same as in Experiment 1, with the four training tasks solved in the first step and the 16 motive-related and neutral tasks solved in the second step. After task completion, a questionnaire was administered to the participants to assess math grade, familiarity with the word problems, or the study on civic competence in numeracy [62], as well as their presumptions of the aim of the study to check their awareness of the experimental manipulation.

### Results and discussion

Again, repeated measures analysis of variance with the four-level within-subjects factor motive imagery was conducted. The dependent variable was the mean number of correctly solved tasks within the four tasks on the same motive imagery (affiliation, achievement, power, and neutral, respectively). Descriptive statistics are presented in Table 4.

**Table 4:** Means and standard deviations for performance in neutral tasks and tasks related to affiliation, achievement, and power in Experiment 2.

	Task performance^1^	
Motive imagery	*M*	*SD*
Affiliation	2.87	0.99
Achievement	3.10	0.84
Power	2.59	1.01
Neutral	2.65	1.08

*Note*.

^1^Number of correctly solved tasks (0–4).

The effect of motive imagery was statistically significant, *F*(3, 186) = 7.03 *p* < .001, η_p_^2^ = .10. Planned simple contrasts showed that the participants solved more achievement-related tasks correctly than neutral tasks, *F*(1, 62) = 16.22, *p* < .001, η_p_^2^ = .21. The difference between affiliation-related and neutral tasks was only marginally statistically significant, *F*(1, 62) = 3.17, *p* = .080, η_p_^2^ = .05, indicating slightly better performance in affiliation-related tasks compared with the neutral ones. There was no statistically significant difference between power-related and neutral tasks *F*(1, 62) = 0.23, *p* = .631, η_p_^2^ = .004. We additionally tested for a possible gender effect. Neither gender nor the gender x motive imagery interaction was statistically significant (*p*s > .10).

Again, we were able to demonstrate that motive imagery in tasks can contribute to better student performance. The results showed a similar pattern as in the first experiment: compared with tasks with neutral topics, the strongest effect appeared for achievement-motive imagery, which was followed by affiliation-motive imagery. No effect was found for power-motive imagery on performance compared with neutral tasks. In the previous experiments, the mathematical word problems within each mathematical topic (e.g., geometry) were comparable when it came to relevant features, such as their mean word account, text complexity, and mathematical operations. However, because of the experimental within-subjects design, we had to use different number values for the tasks belonging to the same mathematical topic (e.g., simple arithmetic). Within a third experiment, we wanted to ensure that the differences between motive-related and neutral tasks could not be explained simply by the different number values in the tasks. Therefore, within the third experiment, we again administered the 16 motive-related and neutral word problems to another sample of students but interchanged the number values in each task that belonged to the same mathematical topic (e.g., simple arithmetic). That is, the task contexts (motive-related or neutral), as well as the mathematical operations, were the same as in the first and second experiments, and only the number values (see Table 1, printed in bold) in the tasks were permuted.

## Experiment 3

The aim of this experiment was to replicate and further substantiate the previous findings for motive imagery in mathematical tasks on students’ task evaluation and performance. For the experiment, we used the same motive-related and neutral word problems as in the previous experiments but randomly permuted numerical data in the tasks on the same mathematical topic (e.g., simple arithmetic) by using a random generator. We did so to ensure that the differences between motive-related and neutral tasks cannot be explained simply by different numerical data (cf. Table 1, bold printed values) in the tasks.

### Methods

#### Participants

Again, the participants were recruited via e-mails, flyers, and notice boards at the university and other public buildings. A total of 67 participants took part in the experiment. We had to exclude five participants from analyses because they had already participated in the first or second experiment. Thus, there were 62 remaining participants (49 female). Their average age was 23.89 (*SD* = 3.10), ranging from 19 to 33 years. All were students at a German university. They studied different subjects. The average last math grade in high school was 2.53 (*SD* = 1.17). They received 10 euros for their participation.

#### Measures and procedure

The main procedure was similar to Experiment 1. However, there were some differences, as described below. Like Experiment 1, the present experiment consisted of two parts: assessing intrinsic task value and self-efficacy expectations in the first part and performance in the second part. In contrast to Experiment 1, the first part was conducted via the internet. We did so for practical reasons so that the participants only had to attend the laboratory once. During the *first part*, the participants answered a short questionnaire with demographics and were then instructed to carefully read and evaluate the subsequent mathematical tasks. Next, the 16 motive-related and neutral word problems were displayed in two blocks, and the participants evaluated each task regarding intrinsic task values (first block) and self-efficacy expectations (second block). Again, the two blocks were balanced: half of the participants started with block A, which measured intrinsic task value, and half of the participants started with block B, which measured task-related self-efficacy expectations. The presentation of the motive-related and neutral math tasks within blocks A and B was randomized (individually for each participant). As in Experiment 1, the intrinsic task value was assessed by the question, “How much do you like the task?” on a 6-point scale ranging from 1 = *very much* to 6 = *not at all*. In contrast to Experiment 1, we decided to assess the participants’ task-related self-efficacy expectations with the following question: “How well do you expect to solve this task?” The participants responded on a 6-point scale ranging from 1 = *very good* to 6 = *not at all*. By using a rating scale with a predefined range, we wanted to reduce strong variations in the data, as was the case in the first experiment. In addition, this item better reflects the corresponding construct of the expectancy-value model of achievement motivation (cf. [21]) at the task level.

*The second part* of the experiment was conducted in a small and separate laboratory cabin. The participants were not allowed to take any belongings (e.g., calculator, smartphone) with them. Before starting the experimental procedure, as described in the previous experiments, the participants completed a computer-driven version of the Multi-Motive Grid (MMG; [69, 70]), which measures the students’ affiliation, achievement, and power-motive dispositions (data for students’ motives are not reported in the present paper because these data are not related to the purpose of the study). The MMG was followed by a motive-neutral filler task to avoid carry-over effects. Therefore, abstract paintings with cubist forms and that were not related to motive imagery were subsequently presented on the computer, and the participants indicated how much they liked the pictures. Next, the participants were instructed to solve motive-related and neutral word problems. This procedure was the same as in the previous experiments, with completion of the four training tasks in the first step, followed by completion of the 16 motive-related and neutral tasks. After task completion, we additionally assessed the participants’ math-related self-concept and math anxiety to detect the possible relations between self-efficacy expectations, task values, and performance with more general ability beliefs (cf. [71]). Therefore, we administered two scales from the international student questionnaire from PISA 2012 [72] to assess students’ math-related self-concept and math anxiety with five items (math anxiety, e.g., “I get nervous solving mathematical problems”; math self-concept, e.g., “I’ve always thought mathematics was one of my best subjects”) [72]. The participants answered on a 4-point Likert scale (1 = *completely true* to 4 = *not true at all*). For math anxiety, Cronbach’s α was .89 (*M* = 2.53, *SD* = 0.82), and for math-related self-concept, Cronbach’s α was .92 (*M* = 2.60, *SD* = 0.85). Finally, the students noted their last math grade in high school, familiarity with the word problems or the study on civic competence in numeracy [64], and their presumption of the aim of the study to check their awareness of the experimental manipulation.

### Results and discussion

Data analysis was analogous to Experiment 1. The dependent variables were the mean liking ratings (intrinsic task value), the mean self-efficacy rating (self-efficacy expectations), and the mean number of correctly solved tasks (performance) in the four tasks on the same motive imagery (affiliation, achievement, power, and neutral), respectively. The Greenhouse–Geisser adjustment was used to correct for violations of sphericity and will be indicated in the corresponding sections, if needed. Descriptive statistics are presented in Table 5. Additional analyses for possible gender effects revealed no gender main effect or gender x motive imagery interaction on task evaluation (intrinsic task value, self-efficacy expectation) or performance (*p*s > .10).

**Table 5:** Means and standard deviations for intrinsic task values, expectancies of success and performance in neutral tasks, and tasks related to affiliation, achievement, and power in Experiment 3.

	Intrinsic task values^1^		Expectancies of success^1^		Task performance^2^	
Motive imagery	*M*	*SD*	*M*	*SD*	*M*	*SD*
Affiliation	4.33	0.97	4.79	1.00	2.53	1.04
Achievement	4.38	0.97	4.80	0.99	2.79	0.93
Power	4.09	1.01	4.70	1.10	2.63	1.10
Neutral	4.21	1.04	4.78	1.00	2.50	1.05

*Note*.

^1^Items were recoded so that high numbers indicate high intrinsic task values (1–6) and high expectancies of success, respectively (1–6).

^2^Number of correctly solved tasks (0–4).

For the correlations between the variables’ intrinsic task value, self-efficacy expectations, and performance, see Table 6. As in the first experiment, correlations were in the expected directions (high intrinsic task values and high task-related self-efficacy expectancies related to better task performance). The same holds for the relations between math anxiety and math-related self-concept with task-related values and expectancies, as well as with task performance. A high math-related self-concept was associated with better task performance, higher intrinsic task values, and task-related self-efficacy expectancies. For math anxiety, it was the opposite (cf. Table 6).

**Table 6:** Intercorrelations for students’ math grades in high school, math-related self-concept, math anxiety, with intrinsic task values, expectancies of success and performance in neutral tasks and tasks related to affiliation, achievement, and power in Experiment 3.

		Math. Self- concept^2^	Math anxiety^2^	Intrinsic task values^3^				Expectancies of success^3^				Task performance^4^			
				neutr.	aff	ach	pow	neutr.	aff	ach	pow	neutr.	aff	ach	pow
**math grade** ^**1**^		-.66**	.53**	-.22	-.18	-.12	-.16	-.29*	-.27*	-.26*	-.27*	-.31*	-.47**	-.31*	-.16
**math. self-concept**		−	-.89**	.48**	.38**	.40**	.39**	.62**	.58**	.59**	.57**	.52**	.54**	.38**	.38**
**math anxiety**			−	-.47**	-.37**	-.42**	-.43**	-.66**	-.64**	-.64**	-.63**	-.47**	-.56**	-.37**	-.43**
**intrinsic task values**															
	neutral (neutr.)			−	.90**	.86**	.85**	.63**	.64**	.64**	.61****	.39**	.43**	.25*	.33**
	aff				−	.89**	.88**	.63**	.68**	.67**	.63**	.37**	.40**	.25*	.34**
	ach					−	.85**	.67**	.72**	.74**	.66**	.37**	.37**	.20	.32*
	pow						−	.63**	.71**	.73**	.71**	.42**	.38**	.24	.35**
**expectancies of success**															
	neutr.							−	.94**	.93**	.92**	.53**	.48**	.34**	.53**
	aff								−	.97**	.94**	.49**	.44**	.32*	.48**
	ach									−	.94**	.49**	.38**	.30*	.45**
	pow										−	.54**	.48**	.31*	.46**
**performance**															
	neutr.											−	.63**	.46**	.60**
	aff												−	.58**	.68**
	ach													−	.60**
	pow														−

Note

* p < .05

** *p* < .01

*N* = 62.

^1^ German grading system, where low numbers indicating high grades (e.g., 1 = very good, 6 = very bad).

^2^ Items were recoded so that high numbers indicate high anxiety and high math-related self-concept, respectively.

^3^Items were recoded so that high numbers indicate high intrinsic task values and high expectancies of success, respectively.

^4^Number of correctly solved tasks.

#### Intrinsic task values

Once again, repeated measures analysis of variance revealed a statistically significant main effect for motive imagery *F*(3, 183) = 7.90, *p* < .001, η_p_^2^ = .12. Planned simple contrasts showed that the participants liked achievement-related tasks more than neutral tasks *F*(1, 61) = 5.91, *p* = .018, η_p_^2^ = .09. The difference between the ratings for affiliation-related and neutral tasks *F*(1, 61) = 3.65, *p* = .061, η_p_^2^ = .06, as well as the difference between power-related and neutral tasks *F*(1, 61) = 3.02, *p* = .087, η_p_^2^ = .05, was only marginally significant, indicating that the participants liked affiliation-related tasks more and power-related tasks less compared with neutral tasks.

#### Self-efficacy expectations

Mauchly’s test revealed that the assumption of sphericity had been violated for the factor motive imagery (χ2(2) = 15.92, *p* < .01); therefore, the degrees of freedom were corrected using Greenhouse–Geisser estimates of sphericity (ε = 0.876). Repeated measures analysis of variance revealed no statistically significant main effect for motive imagery, *F*(2.628, 160.284) = 1.88, *p* = .142, η_p_^2^ = .03.

#### Performance

Regarding the mean number of correctly solved tasks, repeated measures analysis of variance revealed only a marginally statistically significant main effect for motive imagery *F*(3, 183) = 2.42, *p* = .068, η_p_^2^ = .04. Planned simple contrasts showed that the participants solved more achievement-related tasks correctly than neutral tasks, *F*(1, 61) = 4.92, *p* = .030, η_p_^2^ = .08. The difference between affiliation-related and neutral tasks *F*(1, 61) = 0.79, *p* = .780, η_p_^2^ = .001 and the difference between power-related and neutral tasks *F*(1, 61) = 1.11, *p* = .297, η_p_^2^ = .02 did not reach statistical significance.

In sum, we could replicate some of the previous findings for the effects of motive imagery in mathematical tasks on students’ task evaluation and performance. What is most interesting is that the achievement imagery in tasks again increased students’ performance, though the effect was less pronounced than in the previous experiments. This time, however, affiliation-motive imagery did not affect students’ performance in the respective tasks. Regarding intrinsic task values, it was again shown that the students liked affiliation-related tasks more than neutral ones, which is in line with Experiment 1 and earlier research [62]. In addition, the students liked achievement-related tasks more and power-related tasks less than neutral tasks. Task-related expectancies of success were not influenced by motive imagery.

## Additional analyses

We additionally performed analyses on the pooled data from experiments with performance as the dependent variable (pooled data from Experiments 1, 2, and 3), as well as with intrinsic task value as the dependent variable (pooled data from Experiments 1 and 3). In doing so, we included a variable representing the effect of the experiments to further investigate if there are significant differences between the experiments regarding students’ task evaluation (intrinsic task value) and performance. The task-related success expectancies in Experiments 1 and 3 were measured differently (see p. 19 and p. 31). Because these variables are not comparable, we do not provide analysis of the pooled data for success expectancies.

### Performance

Repeated measures analysis of variance with the four-level within-subjects factor motive imagery and three-level between-subjects factor experiment was used to analyze the pooled data from Experiments 1–3. The dependent variable was the mean number of correctly solved tasks (performance) in the four tasks on the same motive imagery (affiliation, achievement, power, and neutral). The Greenhouse–Geisser adjustment was used to correct for violations of sphericity and will be indicated, if needed. The descriptive statistics are presented in Table 7.

**Table 7:** Means and standard deviations for performance in neutral tasks and tasks related to affiliation, achievement, and power (pooled data from Experiments 1, 2, and 3).

	Task performance^1^	
Motive imagery	*M*	*SD*
Affiliation	2.76	1.01
Achievement	2.97	0.89
Power	2.60	1.03
Neutral	2.56	1.06

*Note*. *N* = 183.

^1^Number of correctly solved tasks (0–4).

Repeated measures analysis of variance revealed a statistically significant main effect of motive imagery *F*(3, 540) = 15.17, *p* < .001, η_p_^2^ = .08. Planned simple contrasts showed that the participants solved more affiliation-related tasks correctly than neutral tasks, *F*(1, 180) = 9.63, *p* = .002, η_p_^2^ = .05. The same applied for the difference between achievement-related and neutral tasks *F*(1, 180) = 35.54, *p* < .001, η_p_^2^ = .17. The difference between power-related and neutral tasks did not reach statistical significance, *F*(1, 180) = 0.21, *p* = .644, η_p_^2^ = .001. In addition to the main effect for motive imagery, there were no other statistically significant effects. Neither the main factor experiment (*F*(2, 180) = 0.91, *p* = .403, η_p_^2^ = .01) nor the experiment x motive imagery interaction (*F*(6, 540) = 1.76, *p* = .105, η_p_^2^ = .02) reached statistical significance. That is, there were no significant differences between the three experiments regarding students’ performance in motive-related and neutral tasks.

### Intrinsic task values

In line with previous analyses on pooled data, repeated measures analysis of variance with the four-level within-subjects factor motive imagery and the two-level between-subjects factor experiment was used to analyze pooled data from Experiments 1 and 3. The dependent variable was the mean liking rating (intrinsic task value) for the four tasks on the same motive imagery (affiliation, achievement, power, and neutral). The Greenhouse–Geisser adjustment was used to correct for violations of sphericity and will be indicated, if needed. The descriptive statistics are presented in Table 8.

**Table 8:** Means and standard deviations for the intrinsic task values for neutral tasks and tasks related to affiliation, achievement, and power (pooled data from Experiments 1 and 3).

	Intrinsic task value^1^	
Motive imagery	*M*	*SD*
Affiliation	4.43	0.85
Achievement	4.43	0.84
Power	4.11	1.03
Neutral	4.29	0.91

*Note*. *N* = 120

^1^Items were recoded so that high numbers represent high intrinsic task values (1–6).

Mauchly’s test revealed that the assumption of sphericity had been violated for the factor motive imagery (χ2(2) = 17.199, *p* < .01), which means the degrees of freedom were corrected using Greenhouse–Geisser estimates of sphericity (ε = 0.910). Repeated measures analysis of variance revealed a statistically significant main effect for motive imagery *F*(2.731, 322.278) = 18.25, *p* < .001, η_p_^2^ = .13. Planned simple contrasts showed that the participants liked affiliation-related tasks more than neutral tasks, *F*(1, 118) = 9.58, *p* = .002, η_p_^2^ = .08. The same was true for the difference between achievement-related and neutral tasks *F*(1, 118) = 7.61, *p* = .007, η_p_^2^ = .06. In contrast, the participants liked power-related tasks less than neutral tasks *F*(1, 118) = 10.50, *p* = .002, η_p_^2^ = .08. Neither the main factor experiment (*F*(1, 118) = 0.80, *p* = .373, η_p_^2^ = .01) nor the experiment x motive imagery interaction (*F*(2.731, 322.278) = 1.16, *p* = .325, η_p_^2^ = .01) reached statistical significance. Thus, there were no significant differences between the first and third experiments in terms of students’ ratings of the attractiveness/intrinsic value of motive-related and neutral tasks.

## General discussion

### Summary and interpretation of the main results

In the three experiments, we found positive effects of motive imagery in mathematical word problems on students’ task performance. In particular, this applied to achievement-related tasks, which were superior to neutral ones in all of the experiments. This was also true after the numbers in the tasks on the same mathematical topic (e.g., simple arithmetic) had been randomly permuted. However, regarding the effect sizes, the positive effect weakened across the three experiments. Although in the first experiment we found considerably strong effects of achievement imagery on performance, the effect was rather small in the third experiment. The same applied for the positive effect of affiliation-related tasks on students’ performance. In the first and second experiments, we found that students performed better in tasks relating to affiliation-motive imagery than neutral tasks. This effect was less pronounced in the second experiment. The differences in task performance between motive-related and neutral tasks could also stem from the differences in the difficulty of numerical data in the tasks, so we randomly permuted numerical data within the third experiment. Nevertheless, we subsequently analyzed task difficulties by using the resolution rates of all 16 tasks across the three experiments. When comparing the resolution rates of all tasks in Experiments 1 and 2 with those from Experiment 3 (in which the numerical data were randomly permuted), we found an indication that numerical data of one mathematical topic (direct and inverse proportionality) may have influenced task performance in affiliation-related tasks in the third experiment (S1 File resolution rates). At the same time, the positive influence of achievement imagery on task performance seemed to be robust because the numerical data in the three experiments did not determine solution rates in the achievement-related tasks (S1 File resolution rates).

Within the first and third experiments, we additionally assessed students’ task-related expectancies and values. In both of the experiments, the motive imagery affected students’ intrinsic task values. The pattern of results was similar, showing that the students liked affiliation- and achievement-related tasks more and power-related tasks less compared with the neutral ones. Except for the negative evaluation of power-related tasks, these findings are comparable to prior research findings [60], in which students have preferred particular affiliation-related tasks, followed by achievement-related tasks. The pattern also resembles the preferences of personally relevant issues for adolescents and young adults [54, 55] who prefer social issues, followed by achievement-related issues, as well as power- and prestige-related issues. In contrast to our expectations, motive imagery did not consistently affect students’ task-related expectancies. Although in the third experiment no effect was shown, motive imagery (power imagery) even negatively affected students’ task-related self-efficacy expectations in the first experiment. However, these results are not fully comparable because the students’ self-efficacy expectations were measured differently in the first and third experiments. Regarding power imagery, it turned out that these topics not only decreased students’ task-related expectancies, but also intrinsic task values. These results are somewhat surprising because other research has revealed that adolescents and young adults rated power- and prestige-related issues as important, too [54, 55]. In addition, earlier experiments with school students showed positive effects for power topics in tasks on students’ task evaluation, albeit less consistent and less pronounced than for achievement and affiliation topics [60]. However, these former experiments were conducted with fifth-grade students in their early adolescent years. Thus, the negative evaluation of power-related tasks in the present experiments could be because of the decreasing importance of power issues during adolescence and young adulthood. Even though research has shown that adolescents and young adults consider power- and prestige-related topics to be important [54, 55], it was also revealed that these issues have lost importance on young people in recent years when compared with achievement- and affiliation-related issues [54, 73]. In line with this, Emmons [74] found that personal strivings relating to the power motive were reported most scarcely by undergraduate students when compared with personal strivings relating to affiliation and achievement motives. However, this still does not fully explain why the students in the present study rated power-related tasks as even less attractive than neutral tasks. Another reason for the negative ratings of power-related tasks could be that power-related task contexts (i.e., power imagery) are potentially negatively connoted. Our aim was to use formulations with comparable emotional connotations. However, the participants may have perceived some power-related tasks negatively, for instance, when it was about a person teasing someone else or convincing and persuading other people. In this context, social desirability may also have influenced students’ task evaluation. According to Winter [59], the power imagery in a text is also expressed when a text contains issues about triggering positive emotions in other persons or groups or by controlling or influencing other people, groups, or institutions. Perhaps, only power-related formulations without devaluating or teasing others should be used by speaking of “inspiring other people” or “to move things in a positive direction,” rather than just exerting control [59, pp. 15–20]. Tasks referring to these topics are potentially more positively connoted and could reduce the potential problem of social desirability. Future studies would be necessary to clarify if such power-related topics have the potential to positively affect task-related expectancies and values or if they are detrimental to students’ motivation.

Based on the present experimental results with young adults, power imagery does not seem to be suitable for making tasks more appealing. However, compared with neutral tasks, power imagery was neither beneficial nor detrimental for students’ performance. For affiliation and achievement imagery, the present results further underpin earlier research results [60], indicating that achievement and affiliation topics are personally appealing for students and have the potential to increase students’ intrinsic task values.

#### Relations between students’ task evaluation and task performance

The data from the first and third experiments gave us the opportunity to examine the relationships between students’ task evaluation (task-related expectancies and values) and performance in the corresponding tasks. According to the expectancy-value model and related findings, increased intrinsic task values should lead to increased motivation to engage in the corresponding tasks, thus potentially influencing performance [19–21]. Following this, one could assume that students performed better in those tasks to which they were more attracted. The relations between students’ task values and performance were in the expected directions (albeit mostly insignificant in the first experiment), showing that high intrinsic task values are related to better task performance. However, these relations apply to all tasks and do not show specific links between the evaluation of certain motive-related tasks and performance in the corresponding tasks. In other words, a high intrinsic task value is related to good task performance in general. Thus, it may also be that students who are more competent in mathematics are also more interested in mathematics (e.g., [15]); consequently, these students would assign higher intrinsic value to the math tasks in the present study. The same holds for task-related expectancies. The present correlational findings between participants’ math grade, math self-concept, and math anxiety with participants’ task-related expectancies, values, and task performance (see Table 6) further indicate that students’ mathematical competencies and more stable ability beliefs, such as their math self-concept and math anxiety, played a crucial role in task evaluation and performance within the present study. These relations would correspond to the expectancy-value model, in which expectancies and values are assumed to be influenced by the ability self-concept [20, 21]. Accordingly, other research findings have shown positive relations of prior math grades and students’ math self-concept with students’ math achievement (e.g., [71, 75]), as well as negative relations of students’ math anxiety with math achievement (e.g., [76, 77]).

However, it should be noted that higher task values do not necessarily directly need to influence performance in the respective tasks and, nevertheless, could promote learning. Research has shown that subjective task values are stronger predictors of intentions to engage in math and academic career choices, such as course plans and enrollment decisions than predictors of performance or grades (see [20, 21, 29, 78]). In addition, increased intrinsic task value has the potential to increase students’ enjoyment (cf. [79]) and is associated with the development of more stable personal interest, which is beneficial for students’ learning and performance in the long term [15–17, 79–82]. In the present study, the students assigned higher intrinsic task values for affiliation- and achievement-related tasks than for neutral tasks that did not deal with these issues. If the students feel personally connected to those mathematical problems, they would more likely to be motivated to deal with them. Therefore, selecting tasks referring to affiliation and achievement imagery could be worthwhile for students’ motivation and learning over time.

### Limitations and future directions

Turning to the direct impact of motive imagery in tasks on students’ performance and its possible underlying mechanisms, we assume that motive imagery makes tasks more appealing because of its personal relevance, leading to affective involvement and increased effort and persistence in task completion, thus increasing performance [24, 28, 40]. Such underlying mechanisms cannot be verified by the present findings because we did not measure the participants’ affective involvement during the process of task solving. Future studies are necessary to investigate this issue. Given the present findings for achievement imagery in math tasks, the positive effect on students’ performance could also be because of a semantic achievement priming effect [65, 83, 84]. In the achievement domain, it was found that the presentation of achievement-related words like “master,” “succeed,” or “win” led to higher performance compared with the presentation of neutral words without such achievement primes [83]. Semantic achievement primes are assumed to be connected to previous achievement goals. Reading achievement primes will unconsciously activate these goals (such as the goal to perform well) and guide behavior according to the goal, hence leading to higher performance in a subsequent task [83]. The semantic achievement priming effect was also found for achievement primes in schoolbook texts (math and language arts textbooks), which represent a natural priming context [65]. Including achievement-related words (achievement primes) into the running text is quite comparable to our approach of enriching word problems with achievement imagery. Indeed, across our three experiments, when compared with neutral ones, we found the most reliable effect results on students’ task performance for achievement-related word problems. It cannot be ruled out that the same mechanism—semantic achievement priming—could be applied for achievement-related word problems in the present experiments.

According to our initial assumption that motive imagery could increase performance because of its personal relevance leading to affective involvement, one might have expected more consistent results for affiliation imagery on students’ task performance. After all, social issues are personally relevant for young people (cf. [1, 2, 7, 54, 55]). Nevertheless, it is likely that some other personal factors additionally influenced students’ task performance. It is known that students’ performance in contextualized math problems also depends on their individual characteristics, such as arithmetic skills and text comprehension [85], as well as other cognitive factors such as working memory or inhibitory control [43, 86]. We did not control for these individual cognitive variables, which should be considered in future studies. In addition, it is possible that the students’ personal motives contributed to the process of task completion, which should be further investigated. Because a within-subject design is probably not appropriate for this purpose, we would pursue this idea in upcoming studies by implementing a between-subjects design. In this way, students would be exposed to only one condition (tasks relating to only one motive-related topic) to avoid the carry-over effects of motive imagery in tasks. In doing so, it would be interesting to further investigate the underlying process of task processing by measuring students’ affective involvement, effort, and persistence during task completion and to clarify if students’ motives additionally contribute to these processes.

Another limitation of the present experiments is that we only used a certain type of mathematical task: “dressed-up” word problems (cf. [41, 42]). Dressed-up word problems are mathematical tasks that are only “dressed up in a figurative context” [87, p. 145]. In these problems, the relevant data for finding the solution are already given in the text, and students do not need to make assumptions about missing data [42]. By using only one type of math problem, we wanted to ensure that the problem types were comparable. Even though dressed-up word problems do have their benefits depending on their purpose and are used in math lessons and textbooks (cf. [42, 43, 87]), they represent only a part of the mathematical problem types in mathematics instruction. There has been a critical debate in mathematics education on the practice of using such “artificially” dressed-up word problems in math lessons because they may not foster students’ competencies for mathematical modeling and applied problem solving (cf. [43, 87–89]). The current practice in mathematics education focuses more on the use of realistic, authentic problem types, which are better suited for enhancing students’ mathematical modeling skills. In view of its practical relevance, future research should also examine the influence of motive imagery in modeling problems on students’ task performance and task-related success expectancies and intrinsic values. Solving modeling problems requires a more demanding transfer process between the real world and mathematical world and, thus, more demanding cognitive activities (e.g., [42]). The use of more complex problems in experimental settings would also have the advantage that task completion could depend even more on motivational factors and, to a lesser extent, on students’ prior mathematical knowledge or basic mathematical skills. Hence, the effects of motive imagery in tasks on students’ performance with its underlying mechanisms (e.g., affective involvement, effort, persistence) could be examined more thoroughly.

### Practical implications

Even though the word problems in our present study represent only a part of the word problems used in mathematics instruction, they do play a role in mathematics instruction. Furthermore, schoolbook analyses have shown that motive imagery is used for contextualizing mathematical word problems [59]. In addition, there is some evidence that motive-related topics in tasks and textbooks are biased based on age stereotypes. Whereas affiliation imagery was mainly found in the tasks and textbooks for children in primary school, achievement and power imagery dominated in secondary schoolbooks [59]. Within the present study, it was again shown that affiliation imagery and achievement imagery can enhance students’ intrinsic task value, which is an important aspect in the expectancy-value model of achievement motivation [20, 21]. The present study was conducted with young adults, indicating that affiliation-related issues should be attractive not only for primary school students, but also for older students. Moreover, the present study extended previous research on motive imagery in tasks [60] by showing that in particular, achievement imagery increased students’ task performance. There is also some evidence for the positive effects of affiliation imagery on students’ performance that needs to be further examined. Given the present and previous results, affiliation and achievement imagery could be used for selecting tasks that are more appealing for students, which could be beneficial for their motivation and learning in the long term. In addition to individually personalizing tasks (e.g., with various interests), this could be a practical approach for selecting or developing problems that students are more attracted to and, therefore, likelier to be motivated to deal with.

## Supporting information

S1 File Resolution rates of all tasks in Experiments 1–3. (XLSX)
